# Insights from a multinational survey on ERC courses: a cross-sectional analysis of participant and instructor perspectives

**DOI:** 10.1016/j.resplu.2026.101379

**Published:** 2026-06-08

**Authors:** Vlasios Karageorgos, Timo de Raad, Sander van Goor, Francesc Carmona Jimenez, Andrew S. Lockey, Robert Greif, Carsten Lott, for the Course Strategy Taskforce, John Madar, Jana Djakow, Silvija Hunyadi-Anticevic, Patricia Conaghan, Nikolaos Nikolaou, Joachim Schlieber, Jacques Delchef, Marta Przybyl, Tanya Esposito, Elizabete Neutel, Kevin Mackie, Sabine Nabecker, Abel Roland, Marlie Van Gils

**Affiliations:** a Cardiopulmonary Resuscitation Lab, School of Medicine, University of Crete, Heraklion, Greece; b Department of Anaesthesiology, Onassis Hospital, Athens, Greece; c Department of Pediatric Intensive Care, University Medical Centre Utrecht, Utrecht, the Netherlands; d Emergency Medical Services, RAV Haaglanden, The Hague, Netherlands; e Sistema d’Emergències Mèdiques, Barcelona, Spain; f School of Human and Health Sciences, University of Huddersfield, Huddersfield, UK; g Emergency Department, Calderdale & Huddersfield NHS Trust, Halifax, UK; h Faculty of Medicine, University of Bern, Switzerland; i Kreisverwaltung Mainz-Bingen, Ingelheim, Germany

**Keywords:** Resuscitation education, ERC courses, Assessment, Faculty development

## Abstract

**Background:**

European Resuscitation Council (ERC) life support courses are delivered internationally to standardise resuscitation. Given advances in educational science, evolving learner needs, and rapid technological innovation, ongoing evaluation is required to ensure these courses continue to meet expectations. This study aimed to identify priorities for course delivery, teaching and assessment strategies, and to inform future course development.

**Methods:**

From May to June 2025, the ERC Course Strategy Taskforce conducted two web-based surveys among course participants and instructors (10 and 22 items, respectively). The surveys included single-response items, Likert-scale ratings, and open-text questions addressing teaching background, course delivery, assessment practices, and views on course structure and materials. Data were analysed using a mixed-methods approach, with descriptive statistics applied to quantitative data and inductive thematic analysis to qualitative responses.

**Results:**

Responses from 13,989 participants and 1923 unique instructors across all ERC course types were analysed. Participant satisfaction was high (mean 9.13/10); 92.8% reported increased confidence in real-life resuscitation, and 99.5% rated the on-site component positively. Instructors favoured continuous assessment for evaluating technical skills (95.2%), non-technical skills (92.5%), and professional attitudes (96.2%), with 87.1% supporting its combination with a summative endpoint. Pre-course multiple choice question testing (85.7%) and mandatory instructor preparation (90.8%) were widely endorsed. Qualitative findings highlighted the need to further develop the ERC Course System, expand specialised content, and enable more personalised delivery formats.

**Conclusion:**

ERC courses are highly valued and should evolve towards competency-based assessment, structured faculty development, and enhanced digital infrastructure to maintain relevance, inclusivity, and educational impact.

## Introduction

High-quality resuscitation training is essential to improving outcomes after cardiac arrest and other life-threatening emergencies.^1^ The European Resuscitation Council (ERC) has developed several standardised life-support courses covering the entire spectrum of educational needs in neonatal, paediatric, and adult resuscitation, including Newborn Life Support (NLS), Basic Life Support (BLS) and Paediatric Basic Life Support (PBLS), Immediate Life Support (ILS) and European Paediatric Immediate Life Support (EPILS), Advanced Life Support (ALS) and European Paediatric Advanced Life Support (EPALS).^2^ To ensure consistent instructor competence and faculty development across all courses, the ERC also established the Basic Instructor Course (BIC) for its basic courses and the Generic Instructor Course (GIC) for its advanced courses.

The medical content of these ERC courses is updated every five years in accordance with the most recent published ERC Resuscitation Guidelines.^2^ However, the overall educational approach has remained largely unchanged over the past decades despite advancements in educational science. For example, there has been a shift in educational science towards a more constructivist and connectivist perspective, wherein learners are regarded as active participants in acquiring their own knowledge, skills, and competencies.^3^ Although ERC courses are widely implemented in numerous countries, feedback from course candidates and instructors has not directly influenced the structure of the ERC courses or their educational strategies.

To address this gap, the ERC Course Strategy taskforce conducted a survey among both course participants and instructors, aiming to identify factors influencing the effective conduct of ERC courses and to derive strategies informed by practice and educational science to enhance course effectiveness and the overall learning experience. This approach offers an opportunity to systematically optimise the structure and educational strategy of ERC courses in accordance with contemporary educational science and stakeholder perspectives. Accordingly, this study aims to examine ERC resuscitation-course perceptions of participants and instructors to identify factors that strengthen ERC education and also areas for improvement. These findings are intended to inform, rather than prescribe, future practice- and educational-science-informed approaches to ERC course design and delivery as part of the planned course update process.

## Methods

The ERC Course Strategy Taskforce developed two surveys, one for ERC course participants (candidates) and another for instructors, to assess their views on the existing course structure and content and identify potential areas for change. The survey was informed by a preliminary pilot study conducted in 2023 among ERC instructors teaching advanced courses, led by two authors (TR and CL). The pilot study tested the survey instrument by assessing the clarity, relevance, and completeness of the items. Following structured discussions within the research team, survey items were iteratively refined and reformulated where necessary, with additional questions added by consensus based on their relevance to planned course development, clarity for multinational respondents, and the avoidance of duplication.

The study was conducted using an anonymous English only online survey of course instructors and participants. No translated versions of the survey instruments were used. For the qualitative analysis, only free-text responses provided in English were analysed; non-English responses, if present, were excluded. No identifiable or sensitive personal data were collected. Based on institutional guidance, formal ethics committee approval was not required for this study.

The participant survey comprised six items addressing course content and structure, as well as four items concerning relevant contextual factors. The instructor survey included four items related to course content and structure, ten concerning assessment methods, five regarding instructor preparation, two pertaining to the functionality of the digital ERC course management platform (CoSy), and four related to relevant contextual and legislative requirements. The complete questionnaires are provided as Supplement A. The response formats for the participant survey consisted of six open-ended questions, ten single-response questions, and two questions requiring ratings on either a 4- or 10-point Likert scale (with 1 indicating the least favourable response and 4 or 10 indicating the most favourable). The instructor survey comprised eight open-ended questions, twelve single-response questions, and thirteen 4-point Likert scale questions.

Invitations to participate in the survey were distributed via electronic mail. All unique registered instructors for any course type (*N* = 35,086) and all participants who attended ERC courses during the 11-week study period (*N* = 55,970) were invited to participate. Because many instructors were registered to teach more than one course type, instructor participation was also described at the instructor-course-type level (46,537 eligible entries) when response rates were stratified by course type. Survey responses were collected anonymously through the Survey Monkey platform (San Mateo, CA, USA) from 1 May 2025 to 19 June 2025. Data were analysed utilising SPSS software (IBM, version 23, Armonk, NY, USA). The results for each item were presented as descriptive statistics, including frequencies, proportions, and mean ± SD. Responses to open-text fields were examined using an inductive thematic analysis approach.^4^ All comments were thoroughly reviewed to ensure data familiarisation, and initial codes were generated directly from participants’ expressed viewpoints without the application of a predefined coding framework, by two authors (VK and TR) independently. Discrepancies were discussed to reach consensus. Recurring themes were identified through an iterative process of comparison and refinement, with themes being derived from patterns that consistently emerged across participants’ responses, thereby accurately reflecting participants’ perspectives as expressed in the data rather than imposed by investigators. The thematic summaries were narratively contextualised and used to enrich the quantitative findings.

## Results

The dataset comprised 13,989 of 55,970 participant respondents (25%) and 1923 of 35,086 unique instructor respondents (5.4%) from 53 countries and multiple course types (BLS, PBLS, ILS, EPILS, NLS, ALS, EPALS, BIC, and GIC). As instructors could teach more than one course type, responses were analysed both at the instructor level and at the instructor-course-type level. Overall, 6431 course-type-adjusted responses were received, corresponding to 13.8% of the 46,537 eligible instructor-course-type entries (Table 1). Instructors represented a wide range of educational experience levels, ranging from instructor candidates (7%) to instructors (89.3%) and educators (3.7%), with a mean resuscitation teaching experience of 8.0 ± 7.4 years. Within the ERC course system, instructors teach at the level of ERC provider courses, whereas educators are senior faculty involved in faculty development activities, including the Generic Instructor Course.

**Table 1:** Participant and instructor responses per ERC course type.

	**Participants**			**Instructors**		
**Course type**	**Responded**	**Attended**	**Response rate %**	Instructor responses*	ERC instructors per course type*	**Response rate %**
BLS	7624	39,254	19.4	1394	16,977	8.2
PBLS	640	3795	16.9	592	5124	11.6
ILS	1958	4294	45.6	1086	8130	13.4
EPILS	279	761	36.7	509	2646	19.2
NLS	351	760	46.2	245	1197	20.5
ALS	1840	4124	44.6	1170	7223	16.2
EPALS	608	1435	42.4	637	3334	19.1
BIC	493	1283	38.4	269	862	31.2
GIC	196	264	74.2	529	1044	50.7

Total Responses	13,989#	55,970	25.0	6431	46,537	13.8

# Duplicates excluded.

* The same instructor may teach multiple course types; counted separately for each course type.

### Participant responses

Overall, participants expressed high satisfaction with the structure and learning objectives of ERC courses (Table 2). A total of 13,487 (96.4%) respondents were satisfied with the quality of the e-learning materials. The on-site course part was positively rated by 13,917 (99.5%) participants, and 11,219 (80.2%) found the course length suitable; the remaining participants were evenly split between preferring shorter or longer formats. Additionally, 12,981 (92.8%) participants reported increased confidence in their ability to perform resuscitation in real-life situations after the course, and 13,387 (95.7%) stated that the course content met their expectations. The average overall satisfaction score on courses was 9.1/10.

**Table 2:** Participant rating on course survey.

**Question**	**Poor**	**Unsatisfactory**	**Good**	**Excellent**
How do you rate e-learning?	168 (1.2%)	334 (2.4%)	6139 (43.9%)	7348 (52.5%)
How do you rate the course manual?	133 (0.9%)	252 (1.8%)	6274 (44.9%)	7330 (52.4%)
How do you rate the on-site part?	13 (0.1%)	59 (0.4%)	2663 (19%)	11,254 (80.5%)
	Not confident at all	Somewhat confident	Confident	Very confident
How confident are you about your ability to perform in practice after this course?	40 (0.3%)	917 (6.6%)	6764 (48.4%)	6268 (44.7%)

The qualitative analysis of open-ended responses (*N* = 1105) supported these findings, as 420 (38%) of the comments underscored positive perceptions of instructors, particularly regarding their professionalism, enthusiasm, and capacity to foster a supportive learning environment. Simultaneously, participants identified various areas necessitating improvement. Common requests included increased practical training and simulation exposure 310 (28%), encompassing more scenario-based learning, repetition, and opportunities to practice leadership skills, as well as paediatric or neonatal resuscitation skills. Additionally, concerns were raised about online learning and assessment procedures 295 (27%), which included ambiguous test questions, limited feedback, and technical or translation-related issues. Structural elements such as course pacing and duration 240 (22%), language accessibility of materials 210 (19%), and organisational or logistical factors 175 (16%) were also recurrent themes (Table 3).

**Table 3:** Thematic analysis of participants free-text responses.

**Theme**	**Description**	N** (%)
Participant general comments (N** = 1105)		
Positive feedback on instructors	Praise for professionalism, enthusiasm, knowledge, supportive learning climate	420 (38%)
Request for more practical training/simulation	More hands-on time, more scenarios, repetition, paediatric/neonatal practice, leadership practice	310 (28%)
Online learning & assessment concerns	Ambiguous test questions, inability to see answers, translation issues in exam, unfair pre-test, technical issues	295 (27%)
Course duration & structure	Course too long/too condensed, split into more days, pacing issues, need more breaks	240 (22%)
Language & translation issues	Manuals/videos not in local language, poor translations, mixed-language materials	210 (19%)
Equipment & simulation resources	Outdated mannequins, limited AEDs, malfunctioning equipment, desire for high-fidelity simulation	160 (15%)
Organisational & logistical issues	Communication, access to e-learning, venue problems, group size, scheduling, certificates	175 (16%)
Instructor behaviour concerns (negative)	Intimidating tone, inconsistency in assessment, perceived unfair evaluation	35 (3%)
Desire for broader accessibility/mandatory training	Course should be mandatory, offered more widely, extended to public/schools	95 (9%)

**Participant Improvement suggestions (*N* = 1147)**		
Language & translation issues	Request for full local-language materials; poor translation of manual/tests/videos	390 (34%)
More practical training/more scenarios	Increase hands-on time; more simulations; more repetition; more individual practice; high-fidelity simulation	260 (23%)
Course duration & pacing adjustments	Split into 2–3 days; shorter days; less cognitive overload; better time allocation	210 (18%)
Online learning & test improvements	Show correct answers; clearer MCQs; reduce length; improve pre-test fairness; feedback after test	190 (17%)
E-learning content refinement	Too long; repetitive – text-heavy; need summaries; more videos; structured layout	170 (15%)
Manual & downloadable materials	Request for printed manual; summary sheets; downloadable algorithms; PDF access	140 (12%)
Course organisation & communication	Better pre-course communication; clearer schedule; certificate access issues; earlier material access	110 (10%)
Instructor-related feedback (minority)	Inconsistency between instructors; attitude concerns; need clearer coaching; fairness in assessment	75 (7%)
Specific content additions	Paediatric trauma; non-technical skills; trauma modules; leadership; airway drugs; ECG focus	95 (8%)
Course frequency/refresher requests	Annual refreshers; booster sessions; follow-up webinars; longer validity	85 (7%)
Logistics & facilities	Venue quality; lunch/coffee; noise; equipment; parking; class size	70 (6%)

ECG: Electrocardiogram, MCQ: Multiple Choice Questions.

^**^Excluding no/none answers.

Suggested improvements (*N* = 1147) reflected these concerns, with a strong emphasis on better translation and localisation of materials 390 (34%), expanded hands-on practice 260 (23%), adjustments to course pacing 210 (18%), refinement of online assessment 190 (17%) and e-learning content 170 (15%). Collectively, these findings indicate that while participant satisfaction is high, targeted refinements in practical training, accessibility, and digital learning design could further enhance the learning experience (see Table 3).

### Instructor responses

Instructor feedback was highly positive overall (Table 4). Most instructors, 1869 (97.2%), agreed that the current ERC course format effectively meets its teaching objectives. Pre-course multiple-choice testing was regarded as an effective method for assessing baseline knowledge by 1648 (85.7%) respondents, supporting its continued use as a preparatory learning and screening tool. Instructors expressed a clear preference for continuous assessment across various competency domains. Continuous assessment was supported by 1830 (95.2%) instructors for technical skills, 1785 (92.5%) for non-technical skills (e.g., communication, teamwork, leadership), and 1849 (96.2%) for attitudes.

**Table 4:** Instructor ratings on survey statements.

**Statement**	**Fully disagree *n* (%)**	**Disagree *n* (%)**	**Agree *n* (%)**	**Fully agree *n* (%)**
Current course format meets teaching objectives	4 (0.2)	50 (2.6)	1042 (54.2)	827 (43.0)
Knowledge is tested effectively by pre-course MCQs	40 (2.1)	235 (12.2)	1256 (65.3)	392 (20.4)
Technical skills are tested by continuous assessment	21 (1.1)	72 (3.7)	988 (51.4)	842 (43.8)
Technical skills are tested by summative assessment	46 (2.4)	406 (21.1)	1120 (58.2)	351 (18.3)
Non-technical skills are tested by continuous assessment	20 (1.0)	118 (6.1)	1039 (54.0)	746 (38.8)
Non-technical skills are tested by summative assessment	106 (5.5)	654 (34.0)	920 (47.8)	243 (12.6)
Attitude/affect is tested best by continuous assessment	17 (0.9)	57 (3.0)	1093 (56.8)	756 (39.3)
Attitude/affect is tested best by summative assessment	131 (6.8)	729 (37.9)	849 (44.1)	214 (11.1)
Overall result should be derived by continuous assessment	46 (2.4)	301 (15.7)	1015 (52.8)	561 (29.2)
Overall result should be derived by summative assessment	101 (5.3)	669 (34.8)	934 (48.6)	219 (11.4)
Overall result should be derived by continuous assessment with a summative endpoint	43 (2.2)	206 (10.7)	915 (47.6)	759 (39.5)
The concept of individual support for course candidates is adequate	16 (0.8)	191 (9.9)	1351 (70.3)	365 (19.0)
Individual support for the course candidates should already start with their online preparation (before the course)	67 (3.5)	617 (32.1)	917 (47.7)	322 (16.7)
The time available for individual support of course candidates is sufficient.	48 (2.5)	488 (25.4)	1214 (63.1)	173 (9.0)
CoSy provides adequate administrative support	29 (1.5)	123 (6.4)	1367 (71.1)	404 (21.0)
CoSy is user-friendly	40 (2.1)	199 (10.3)	1262 (65.6)	422 (21.9)
Instructor preparation should be mandatory	24 (1.2)	152 (7.9)	1050 (54.6)	697 (36.2)

CoSy: Course System.

While summative assessment retained some support, particularly for technical skills, the majority, 1674 (87.1%), favoured a combination of continuous assessment with a summative endpoint.

Coaching or individual course participants’ support was considered adequate as a concept by 1716 (89.3%) instructors, although only 1387 (72.1%) felt that sufficient time was available during the ERC courses for this activity. Coaching starting in the pre-course phase was favoured by 1239 (64.4%) respondents.

Qualitative feedback underscored the necessity for earlier and more systematically organised coaching. Specifically, many respondents requested increased protected time for individual feedback 247 (46%) and smaller instructor-to-participant ratios than the course specific minimum (ranging from 1:3 to 1:8) to improve the quality of coaching. Additionally, 161 (30%) of the comments advocated for support commencing in the pre-course phase, while 129 (24%) emphasised the importance of clearer coaching frameworks and well-defined coaching objectives (Table 5).

**Table 5:** Thematic analysis of responses on individual support/coaching during ERC courses (*N* = 538).

**Theme**	**Description**	*n* (%)*
More time for onsite individual support	Increase/protect coaching time during course; breaks are too short	247 (46%)
Start coaching earlier (pre-course/online phase)	Contact before course; support during online preparation; early coach allocation	161 (30%)
Structure/standardisation of coaching	Clear goals, checklists, scheduled slots, guidance on coaching process	129 (24%)
Preference for 1:1 over group coaching	Short one-to-one meetings preferred; group coaching seen as insufficient	92 (17%)
More resources/smaller groups	More instructors, fewer candidates per instructor, longer course/extra stations	88 (16%)
Faculty development for coaching/feedback	Train instructors in coaching, debriefing, learning conversations	70 (13%)
Online communication tools	Chat through ERC app/WhatsApp/videoconference to enable earlier support	58 (11%)
Targeted/conditional support + feasibility concerns	Support only for those needing it; scepticism about feasibility/benefit	54 (10%)
Strengthen online preparation monitoring	Access to use pretest data; online continuous assessment	51 (9%)
No change/satisfied	No suggestions; coaching adequate or unnecessary	156 (29%)

* Multiple themes could be assigned to a single response; therefore, percentages do not sum to 100%.

Instructors reported positive experiences with administrative and digital support provided by ERC. Most agreed that administrative support 1771 (92.1%) and the CoSy platform's user-friendliness 1684 (88%) were of high quality. Nevertheless, thematic analysis (Table 6) identified several areas for improvement, including platform reliability (e.g. time-tracking errors, glitches, slow performance) 114 (23%), navigation and usability 222 (45%), access to learning materials 138 (28%), enhanced analytics, and progress-tracking tools 109 (22%) (Table 4). Requests for mobile functionality (iOS/Android application or mobile-optimised version for course administration) 59 (12%) and improved communication tools 89 (18%) were also common.

**Table 6:** Thematic analysis of responses regarding CoSy improvement (*N* = 494).

**Theme**	**Description**	*n* (%)*
No changes/satisfied	No suggestions, satisfaction with the current system	237 (48%)
Navigation, usability & certification management	Improvement suggestions on interface usability, workflow simplicity, assessment registration, certificate creation, expiry reminders	222 (45%)
Access to learning materials	Difficulty locating manuals and course materials	138 (28%)
Enhanced Platform reliability	Technical issues, time-tracking errors, glitches, slow performance	114 (23%)
Language & translation quality	Requests for additional languages and improved translation accuracy	124 (25%)
Assessment, dashboards & reporting analytics	Progress tracking, MCQ results, appraisal tools, data export	109 (22%)
Communication & support tools	Messaging, chat functions, support responsiveness	89 (18%)
Content enhancement	More videos, case-based learning, simulations, gamification, additional National Resuscitation Council materials	89 (18%)
Onboarding & reminders	Guidance for first-time users, reminders, clearer expectations for course participants	84 (17%)
Administrative workflow & permissions	More permissions to course directors/instructors and admin management	84 (17%)
Course discovery & booking	Additional functions in course finder, calendar and instructor booking	64 (13%)
Mobile app	Requests for iOS/Android apps	59 (12%)
Instructor pathway & coaching	Instructor selection, feedback transparency, faculty development	49 (10%)

MCQ: Multiple Choice Questions.

* Multiple themes could be assigned to a single response; therefore, percentages do not sum to 100%.

Regarding educational content and delivery, instructors recommended expanding coverage of specialised topics such as paediatric trauma, advanced neonatal resuscitation, and extracorporeal cardiopulmonary resuscitation (ECPR). Additional suggestions included strengthening non-technical skills training 36 (9%), improving simulation realism 28 (7%), and reducing overlap between course levels. While most favoured maintaining the established face-to-face model, a minority 27 (7%) supported more flexible or hybrid formats to improve accessibility for busy clinicians and learners in geographically remote areas (Table 7).

**Table 7:** Thematic analysis of instructor suggestions on course content, learning materials, and delivery.

**Theme**	**Description**	*n* (%)*
**Course revision content suggestions (*N* = 397)**		
Expansion of advanced and special-circumstance content	Neuro emergencies, ECPR, PoCUS, FONA, mechanical CPR, hyperbaric resuscitation, special circumstances	62 (16%)
Reduction or removal of selected topics	Remove ABGs, capnography, ECGs, NLS sections in EPALS, reduce trauma content in EPALS	58 (15%)
Paediatric and trauma scope refinement	Paediatric trauma focus, separate paediatric trauma course, reduce trauma overlap in EPALS	31 (8%)
First aid and basic skills expansion	Bleeding control, first aid, victim transport techniques, gasping recognition	18 (5%)
Reduction of ILS/ALS overlap	Clarify boundaries, remove redundant advanced content from ILS	14 (4%)
Simulation realism and clinical applicability	Higher-fidelity simulation, scenario variation, CPR-ventilation quality feedback devices, VR, gamification	28 (7%)
Non-technical skills (NTS)	Focus on leadership, communication, clinical debriefing	36 (9%)
Educational structure of content delivery	Keep some topics online only (ABGs, capnography), hybrid formats, expert courses	27 (7%)
Flexible/hybrid delivery formats	Blended learning, online preparation, modular content, enhanced VLE/CoSy	27 (7%)
Learning materials and manuals	Need for manuals, updated videos, revised CoSy/VLE materials	35 (9%)
Local adaptation and equity	Local customization, low-resource adaptations	19 (5%)
Translation and language quality	Translation issues, multilingual consistency	19 (5%)

**Learning materials and course delivery (*N* = 892)**		
Interactive, video-based, scenario-driven learning	More interaction, less text, more videos/demos, interactive cases, gamification, simulation scenarios	312 (35%)
Translation and language availability (quality + timeliness)	Faster and higher-quality translations; materials/videos/MCQs in local languages; reduce ambiguity/errors	268 (30%)
Materials structure, clarity, and consistency	Better organisation; reduce overload; align manuals/videos/slides; clearer objectives; fewer contradictions	196 (22%)
Preference for paper/PDF manuals and hybrid learning	Need for printed manuals or downloadable full PDFs to support non-digital learners	161 (18%)
Assessment and MCQ quality	Revise unclear/misleading MCQs; improve translation; provide feedback/rationale; rotate questions; exam revision	178 (20%)
Alignment between online preparation and face-to-face course	Stronger connection between e-learning and onsite practice; clarify workload; mandatory completion before attendance	134 (15%)
Instructor development and educational quality support	Improve instructor manuals; faculty development pathways; guidance on coaching and debriefing skills; clearer instructor evaluation criteria	89 (10%)
Simulation resources, equipment, and realism	Need for high-fidelity manikins/software; realistic cases; equipment availability	54 (6%)
Standardisation across countries/centres	Reduce variation across countries; ensure equal access to materials; consistent course rules/support	71 (8%)
No suggestions/satisfied	No changes suggested; materials adequate	223 (25%)

ECPR: Extracorporeal Cardiopulmonary Resuscitation, PoCUS: Point-of-Care Ultrasound, FONA: Front of neck access, ABG: Arterial Blood Gases, ECG: Electrocardiogram, NLS: Newborn Life Support, EPALS: European Paediatric Advanced Life Support, ALS: Advanced Life Support, ILS: Immediate Life Support, MCQ: Multiple Choice Questions, VR: Virtual Reality, VLE: Virtual Learning Environment.

* Multiple themes could be assigned to a single response; therefore, percentages do not sum to 100%.

Feedback regarding learning resources highlighted the need for more interactive, scenario-based educational materials 312 (35%), improved translation and language availability 268 (30%), and better alignment between online preparation and face-to-face teaching 134 (15%) (Table 7).

Mandatory pre-course preparation for instructors was supported by 1747 (90.8%) respondents, highlighting the importance of regular updates on scientific content, educational methodologies, and standard operating procedures. However, in qualitative feedback, concerns were raised about instructor workload 39 (11%) and the necessity to balance mandatory training with the voluntary nature of faculty engagement. Additional themes included improved communication from the ERC, transparent progression pathways, and consideration of financial and logistical barriers to instructor participation (Table 8).

**Table 8:** Thematic analysis of responses regarding instructor preparation and support (*N* = 371).

**Theme**	**Description**	*n* (%)*
No additional comment/thanks/positive feedback	No suggestions, gratitude, general encouragement	219 (59%)
Instructor preparation format & timing	Preparation duration, onsite vs online, varies by experience	44 (12%)
Instructor workload & retention concerns	Avoid too many mandatory tasks; volunteer burden	39 (11%)
Communication & updates from ERC/CoSy	Need timely updates, newsletters, clear contact/support	33 (9%)
Education Instructor Days (EID) views	Mandatory vs optional; usefulness; provide online alternatives	31 (8%)
Course content/material improvements	Update manuals/e-learning/videos; reduce inconsistencies	29 (8%)
Instructor remuneration	Pay/standardise fees; fairness; support for low-resource settings	27 (7%)
Assessment policy & fairness	Continuous vs summative; bias concerns; mandatory tests	21 (6%)
Digital tools/modernization	App requests; CoSy improvements; BIC in CoSy	18 (5%)
Instructor access & progression opportunities	Instructor Candidate placements, transparency, becoming educator/instructor	17 (5%)
Course costs & affordability	High costs for participants/centres	12 (3%)
Equipment & logistics	Equipment purchasing, merchandise/uniforms	8 (2%)

CoSy: Course System, BIC: Basic Instructor Course.

* Multiple themes could be assigned to a single response; therefore, percentages do not sum to 100%.

## Discussion

The results of this large international survey indicate that ERC courses are highly valued by both participants and instructors. Course participants reported high satisfaction with course structure, learning objectives, and instructor performance, accompanied by gains in confidence for real-life resuscitation. Instructors likewise expressed strong support for the existing ERC course framework, highlighting its international standardisation, clear structure, and overall educational quality. Despite this broad endorsement, both groups identified areas for educational development, particularly in assessment strategies, coaching structures, digital infrastructure, and access to learning materials. These findings demonstrate confidence in the current ERC educational model while providing stakeholder-derived priorities that can inform the ERC Course Strategy Taskforce’s rationale for developing and implementing targeted, practice and educational-science-informed advancements that preserve core strengths and align course delivery with contemporary educational practice.

From the participants’ perspective, ERC courses are perceived as highly valuable, confidence-enhancing educational experiences, with exceptionally high overall satisfaction and perceived readiness for real-life resuscitation. Despite this, learners consistently expressed a desire for increased hands-on exposure and repetition, highlighting the central role of experiential learning in perceived competence development.^5^, ^6^, ^7^, ^8^ Concerns raised by participants primarily related to structural elements, e.g. language accessibility, assessment transparency, pacing, and organisational clarity, rather than to instructor performance. These findings suggest that, while instructional delivery is strongly endorsed, optimisation efforts should focus on managing cognitive load, ensuring equitable access to learning materials, and enhancing formative feedback mechanisms to better support learner autonomy and mastery.

A major point strongly advised from instructors was to adopt continuous, formative, competency-based assessment across all ERC courses. This aligns with established adult learning theories^9^, ^10^ and is supported by evidence showing that iterative feedback improves skill acquisition and retention.^11^, ^12^, ^13^ Mandatory instructor preparation, covering both scientific content and educational methods, was strongly supported by instructors as a means of promoting educational consistency and guideline-based teaching. Although resuscitation-specific evidence on mandatory instructor preparation is non-existent, implementing such mandatory preparation poses practical challenges, particularly in accommodating instructors’ busy clinical schedules and aligning training requirements with existing course timetables. In addition, robust mechanisms would be required to verify participation and to evaluate whether mandatory preparation translates into improved instructor performance and, ultimately, enhanced learning outcomes for course participants.

Instructor preferences differed by course format: advanced courses were more often seen as benefiting from an additional summative assessment, whereas continuous assessment alone was deemed adequate for basic courses. Reported advantages of ERC courses included international standardisation, a clear structure, and high-quality materials. The challenges included course duration and faculty recruitment. Several content gaps and different course formats were reported, such as paediatric trauma treatment and advanced neonatal care, which are important inputs for the future development of ERC courses to meet the needs of course participants. The same applies to incorporating more flexible delivery modalities, such as blended or online learning, where seen as appropriate to further improve the accessibility of ERC courses without compromising educational standards.

Notably, despite a large number of courses being BLS with 16,977 instructors, only 8.2% of them responded to the survey, substantially lower than the percentage of instructors from advanced courses (16–50%) (Table 1). This disparity may reflect lower engagement with such surveys or a reduced perceived relevance of strategic discussions among BLS teaching professionals. Given the foundational importance of BLS training globally,^14^, ^15^, ^16^ targeted engagement strategies may be required to better capture this group's perspectives in future consultations.

The modernization of the ERC Course Management platform has become an overarching priority for instructors. Improvements in system reliability, mobile-first accessibility, analytics functionalities, and transparent progress monitoring for both participants and faculty may collectively diminish administrative workloads, enhance user experience, and facilitate data-driven quality assurance. This aligns with evidence from systematic reviews showing that well-designed learning management systems enhance learner engagement, support performance tracking through learning analytics, and improve the overall quality of digital and blended education.^17^

Several knowledge gaps emerge from this survey and warrant consideration in future ERC course development. First, it remains unclear how ERC courses can maintain the strengths of international standardisation and equity while allowing sufficient inclusiveness and diversity, like local adaptation to language, logistics, and resource context. Second, uncertainty remains about the optimal design of assessment and faculty development strategies, including how continuous and summative assessment should be balanced across course types and whether mandatory instructor preparation improves educational or learner outcomes. Third, the comparatively low engagement of BLS instructors indicates that the perspectives of this large and educationally central faculty group remain insufficiently characterised. Finally, because the main findings are presented primarily in aggregate across course types, they may mask course-specific priorities.

For implementation planning, the survey findings may be prioritised using a staged approach. First, high-frequency and comparatively low-risk improvements that address clarity and accessibility could be considered, including translation quality, MCQ wording, material navigation, and communication. Second, course-type-specific pilots could evaluate assessment and coaching changes, particularly continuous assessment, protected feedback time, and instructor preparation. Third, larger structural or digital-system changes, such as enhanced analytics, mobile access, and flexible or hybrid delivery formats, should be assessed for feasibility, workload, equity, and educational outcomes before broader implementation. This staged approach may support alignment with future ERC Course updates and provide a structured framework for integrating stakeholder feedback into ongoing course development.

The limitations of this survey study include the low instructor response rate and marked variation in response rates across course types, which may threaten internal validity through non-response bias. Self-selection bias is also possible, as respondents may have been more engaged or motivated than non-respondents, and participation may have been influenced by differences in digital access or computer literacy. Incomplete data for some survey items represent an additional limitation. Because the survey relied on self-reported perceptions and confidence, the findings cannot be assumed to reflect objective competence, clinical performance, or patient outcomes. Furthermore, results are presented mainly in aggregate across all course types; therefore, they should be interpreted as descriptive, hypothesis-generating priorities for further development and evaluation rather than as evidence supporting specific course changes. To mitigate potential interpretive bias within the author group, analyses were primarily descriptive, and qualitative coding was performed independently by two authors, with discrepancies resolved by consensus. Clearly, no causal inferences regarding the impact of ERC courses can be drawn from this survey, and the generalisability of the findings is confined to regions where ERC courses are offered. Conversely, the strengths of this study include its large sample size and broad representation across multiple ERC course types and participant countries, providing an extensive overview of stakeholder perspectives within contemporary resuscitation education.

## Conclusion

This survey, which solicited feedback from both participants and instructors of ERC courses regarding their perceptions of the current course formats, demonstrated that ongoing assessment, comprehensive faculty training, and the advancement of digital support serve as practical strategies to enhance the future effectiveness of ERC courses. Broadening the scope to include more advanced critical care topics and providing flexible, tailored delivery formats may further increase the relevance and accessibility of ERC courses. The findings of this survey offer valuable insights for the ERC bodies involved in the continuous improvement of all its various course formats.

## CRediT authorship contribution statement

**Vlasios Karageorgos:** Writing – review & editing, Writing – original draft, Project administration, Methodology, Investigation, Formal analysis, Data curation, Conceptualization. **Timo de Raad:** Writing – review & editing, Writing – original draft, Validation, Project administration, Methodology, Formal analysis, Data curation, Conceptualization. **Sander van Goor:** Writing – review & editing, Methodology, Formal analysis, Conceptualization. **Francesc Carmona Jimenez:** Writing – review & editing, Methodology, Formal analysis, Conceptualization. **Andrew S. Lockey:** Writing – review & editing, Supervision, Methodology, Formal analysis, Conceptualization. **Robert Greif:** Writing – review & editing, Validation, Supervision, Methodology, Formal analysis, Conceptualization. **Carsten Lott:** Writing – review & editing, Supervision, Methodology, Conceptualization. **John Madar:** . **Jana Djakow:** . **Silvija Hunyadi-Anticevic:** . **Patricia Conaghan:** . **Nikolaos Nikolaou:** . **Joachim Schlieber:** . **Jacques Delchef:** . **Marta Przybyl:** . **Tanya Esposito:** . **Elizabete Neutel:** . **Kevin Mackie:** . **Sabine Nabecker:** . **Abel Roland:** . **Marlie Van Gils:** .

## Declaration of competing interest

VK is ERC SEC-IES member; TR is ERC SEC-IES co-chair; SG is ERC SEC-BLS co-chair; FC is ERC SEC-ALS co-chair; AL is the ERC President-Elect, and member of the editorial board of Resuscitation Plus; RG is the ERC Director of Guidelines and ILCOR, and member of the editorial board of Resuscitation Plus; CL is the ERC Director of Training and Education.

## Appendix A Supplementary material

Supplement A European Resuscitation Council (ERC) participant and instructor survey questionnaire used for data collection, including all closed- and open-ended survey items administered to course participants and instructors.
