# Supplementary material for: Insights from a multinational survey on ERC courses: a cross-sectional analysis of participant and instructor perspectives
Source: Resusc Plus. 2026 Jun 8;30:101379. doi: 10.1016/j.resplu.2026.101379 (PMC13320541; doi:10.1016/j.resplu.2026.101379)
Supplement: Supplement A — European Resuscitation Council (ERC) participant and instructor survey questionnaire used for data collection, including all closed- and open-ended survey items administered to course participants and instructors. [file mmc1.docx]

**ERC participant survey**

**Question Title**

* 1. How do you rate the e-learning?

- Excellent
- Good
- Unsatisfactory
- Poor

Comments

**Question Title**

* 2. How do you rate the course manual?

- Excellent
- Good
- Unsatisfactory
- Poor

Comments

**Question Title**

* 3. How do you rate the on-site part?

- Excellent
- Good
- Unsatisfactory
- Poor

Comments

**Question Title**

* 4. How confident are you about your ability to perform in practice after this course?

- Very confident
- Confident
- Somewhat confident
- Not confident at all

Comments

**Question Title**

* 5. Do you have any specific comments or suggestions about this course? (Leave empty if none.)

**Question Title**

* 6. What is your overall satisfaction rating with the course?

| Scale 1 to 10 |  |  |
| --- | --- | --- |

**Question Title**

* 7. Would you allow ERC to contact you regarding your answers here?

Yes

No

If Yes, please enter your e-mail address:

**Question Title**

*8. Did the course meet your expectations in terms of the topics and content covered?

Yes

No

**Question Title**

*9. What do you think of the on-site part duration ?

- Should be shorter
- Should be longer
- Duration is fine
- The duration is fine but time should be re-arranged (Please provide more details)

Comments

**Question Title**

* 10. Do you have any suggestions to improve the course format or the course content to better meet your needs?

Open Text

**ERC Instructor Survey**

1. I teach ERC courses for:

(Dropdown in years)

1. I have the following qualifications (checkbox)

|  | NA | IP | IC | Instructor | CDC | CD | Educator |
| --- | --- | --- | --- | --- | --- | --- | --- |
| BLS |  |  |  |  |  |  |  |
| PBLS |  |  |  |  |  |  |  |
| ILS |  |  |  |  |  |  |  |
| EPILS |  |  |  |  |  |  |  |
| ALS |  |  |  |  |  |  |  |
| EPALS |  |  |  |  |  |  |  |
| ETC |  |  |  |  |  |  |  |
| BIC |  |  |  |  |  |  |  |
| GIC |  |  |  |  |  |  |  |

1. How many courses did you teach in the past 2 years (2023 - 2024) (all types of ERC courses) ?

(open numerical answer)

1. How many courses do you plan to teach in 2025 ?

(open numerical answer)

1. Do you believe the current format of the courses you teach, effectively meets the intended teaching objectives?

- Fully agree
- Agree
- Disagree
- Totally disagree

1. What changes would you like to suggest to the course structure? Please note the course type and the change you would like to suggest .
2. Would you like to suggest any changes to the course content and topics covered within courses? Please indicate objectives you want to add or you want to remove
3. We use different types of assessment. Please rate the following statements (Likert Scale: Fully disagree, Disagree, Agree, Fully agree)

- - Knowledge is tested effectively by pre-course MCQs
- - Technical Skills are tested effectively by formative assessment
- - Technical skills are tested effectively by summative assessment
- - Non-technical skills are tested best by formative (continuous) assessment
- - Non-technical skills are tested best by summative assessment
- - Attitude/affect is tested best by formative (continuous) assessment.
- - Attitude/affect is tested best by summative assessment
- - The overall course result should be derived by formative (continuous assessment)
- - The overall course result should be derived by summative assessment
- - The overall course result should be derived by formative (continuous assessment) with a summative endpoint.

1. Regarding online part required time duration
   1. Should be shorter
   2. Should be longer
   3. Duration is fine
   4. Other suggestion (please specify)
2. Regarding course duration of the onsite part
   - Should be shorter
   - Should be longer
   - Duration is fine
   - The duration is fine but time should be re-arranged
3. In your instructor role you support candidates individually throughout the course (Mentoring/Coaching). Please rate the following statements: (Likert Scale: Fully disagree, Disagree, Agree, Fully agree)

- The concept of individual support for course candidates is adequate.
- Individual support for the course candidates should already start with their online preparation (before the course)
- The time available for individual support of course candidates is sufficient.

1. What changes do you suggest to individual support? (Open question)
2. Please rate the following statements: The ERC course management system CoSy: (Likert Scale: Fully disagree, Disagree, Agree, Fully agree)

- provides adequate administrative support

- is user-friendly

1. What changes do you suggest on CoSy environment ? (open question)
2. What changes do you suggest on learning & course materials ? (open question)
3. Further comments to improve our courses. (open question)
4. How do you receive up to date information from ERC/NRC?

- ERC Newsletter

- NRC information

- Eductional Instructor days

- other (please specify)

1. Please rate the following statement: Instructor preparation should be mandatory

(Likert Scale: Fully disagree, Disagree, Agree, Fully agree)

18.What is the minimum timing for the online instructor preparation (short text)

19.What is the maximum timing for the online instructor preparation (short text)

20. What is the minimum timing for the on-site instructor preparation (short text)

21. What is the maximum timing for the on-site instructor preparation (short text)

22. Any additional comments ?
